# Spinal Phlegmon Secondary to Neisseria meningitidis Meningitis With Possible Herpes Simplex Virus 1 Co-infection in an Immunocompetent Pediatric Patient

**DOI:** 10.7759/cureus.80585

**Published:** 2025-03-14

**Authors:** Peter Paul Lim, Kayla Morisette, Meredith Keith, Eric Hines, Mark K Huntington

**Affiliations:** 1 Department of Pediatrics, Division of Infectious Diseases, Avera McKennan University Health Center, Sioux Falls, USA; 2 Department of Pediatrics, Division of Infectious Diseases, University of South Dakota Sanford School of Medicine, Sioux Falls, USA; 3 Department of Family Medicine, University of South Dakota Sanford School of Medicine, Sioux Falls, USA; 4 Department of Family Medicine, Center for Family Medicine, Sioux Falls, USA

**Keywords:** herpesvirus, meningitis, neisseria, pediatrics, phlegmon

## Abstract

Spinal phlegmon has rarely been reported in meningococcemia. Simultaneous polymicrobial infection has been occasionally reported as the etiology of meningitis. The development of a spinal phlegmon and optic neuritis complicated meningitis in a five-year-old male who presented with clinical sepsis and meningitis. *Neisseria meningitidis* (*N. meningitidis*) and herpes simplex virus 1 (HSV-1) were demonstrated in the cerebrospinal fluid. Investigation of potential immunocompromise revealed no identified deficit. Protracted antibiotics and antiviral therapy were completed with a favorable clinical outcome. This is potentially the first reported case of spinal phlegmon associated with meningococcal meningitis in an immunocompetent pediatric patient. It occurred in the clinical context of what appeared to be a simultaneous infection by *N. meningitidis* and HSV-1.

## Introduction

Meningitis is an inflammation of the lining surrounding the brain and spinal cord due to an infectious process. While fungal and parasitic causes exist, viruses and bacteria are the main pathogens. The most common etiology is viral infection; the most severe is bacterial infection [1].

A wide range of viruses may cause meningitis, including herpesviruses, enteroviruses, adenoviruses, arboviruses, lymphocytic choriomeningitis virus, and mumps virus. For a majority of these, treatment is primarily supportive, though antiviral drugs have an important role in some, especially in herpesvirus infection.

Bacterial causes of meningitis include the genera of *Enterobacter*, *Escherichia*, *Haemophilus*, *Listeria*, *Mycobacterium*, *Neisseria*, *Orientia*​​​​​, *Pseudomonas*, *Salmonella*, *Staphylococci*, and *Streptococci*. These may be rapidly progressive, complicated by abscess formation, and may lead to sepsis. Early diagnosis and antibiotic treatment are critical.

Herpes simplex virus (HSV) is one of the most common viral etiologies of meningitis; type 2 is more commonly associated with meningitis than type 1. Transmission is generally via mucosal contact (genital or oral). Long-term neurological sequelae are more common with HSV than in other viral meningitis cases, and it may cause recurrent aseptic meningitis (Mollaret’s meningitis). Early diagnosis and antiviral treatment greatly improve outcomes [2,3].

*Neisseria meningitidis (N. meningitidis*), also known as meningococcus, is a Gram-negative diplococcus that causes severe meningitis and septicemia. It may progress rapidly to death. It is spread by exposure to respiratory and throat secretions, including those of asymptomatic carriers. Age groups with higher rates of meningococcal disease include infants in their first year of life, those ages 16-23 years old, and those over age 85 years old. Individuals with asplenia, human immunodeficiency virus (HIV) infection, or complement deficiencies or those on complement pathway inhibitors are significantly at risk for infection [1].

Meningitis occurring coincident with other systemic infections by a different organism is not rare, but coinfection of the cerebrospinal fluid (CSF) by multiple pathogens as a cause of meningitis is uncommon. These polymicrobial infections are usually associated with neurosurgical procedures, but community-acquired cases, including bacterial-viral coinfections, have been reported [4]. Coinfection with *Neisseria* and HSV has not been previously reported.

We present a case of meningitis with phlegmon attributable to coinfection by *N. meningitidis* and HSV.

## Case presentation

A five-year-old male with a history of speech delay presented to a stand-alone emergency department with an approximately four-day history of decreased energy, non-bilious, non-bloody emesis, loose stools, and fevers followed by acute development of rash in the back and lower extremities, along with subsequent confusion and somnolence.

**Figure 1:**
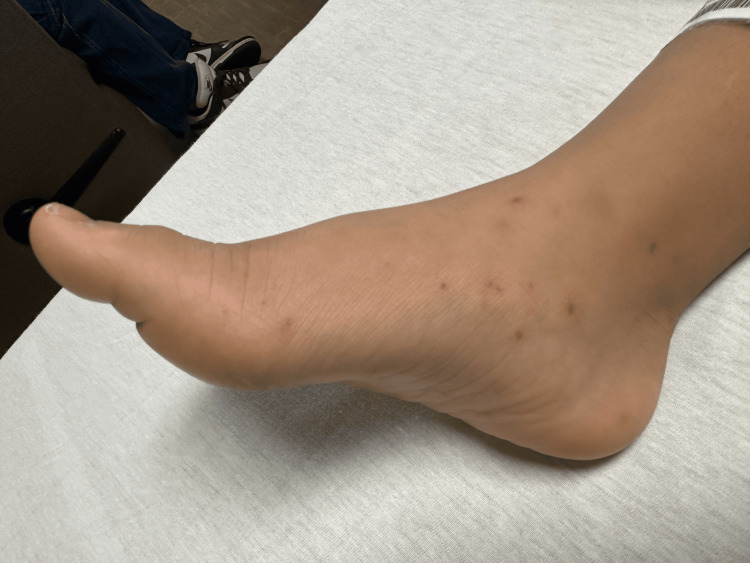
Purpuric rash

On initial examination, his vital signs showed a temperature of 98°F, pulse 101, respiratory rate 20, blood pressure 105/70 mmHg, and room air oxygen saturations of 100%. He was irritable, ill-appearing, and lethargic, with altered mental status and difficulty following commands or responding to questions. Cranial nerves II-XII were intact, pupils sluggish in response to light, no papilledema. There was subtle, non-rigid flexion of the upper extremities and extension of the lower extremities. The deep tendon reflexes were intact. Several petechial to purpuric lesions were noted on his face, back, and lower and upper extremities with involvement of the soles of his feet (Figure 1). He was also noted to have nuchal rigidity. Laboratory evaluation showed a normal leukocyte (WBC) count but predominance of segmented leukocytes, thrombocytopenia, hypokalemia, hyperbilirubinemia, and significantly elevated inflammatory markers (Table 1).

**Table 1:** Summary of pertinent laboratory and imaging data performed throughout the patient’s admission course * drawn after initiation of antibiotics, ** performed twice with same results RBC: red blood cell, CSF: cerebrospinal fluid, PCR: polymerase chain reaction, HSV-1: herpes simplex virus 1, BUN: blood urea nitrogen, ALT: alanine transaminase, AST: aspartate transaminase, ALP: alkaline phosphatase, CRP: C-reactive protein, CT: computed tomography, MRI: magnetic resonance imaging, MRA: magnetic resonance angiography, MRV: magnetic resonance venography, WBC: white blood cell, HHV-6: human herpesvirus 6

Hospital day collected	Source	Test	Results (normal)	Comments
1	CSF	Erythrocyte count (RBC)	5750/uL	Fluid was yellow and cloudy
		Nucleated cells	13500/uL (<7)	
			96% neutrophils	
			4% lymphocytes	
		Glucose	<10 mg/dL (40-70)	
		Total protein	421 mg/dL (40-70)	
		Gram stain	Gram negative diplococci	
		PCR	Positive for *Neisseria meningitidis*	
			Positive for HSV-1	
		Culture	No growth*	
	Blood	Leukocyte count (WBC)	8.5 K/uL (5-15.5)	-
			91.6% neutrophils (38.2-45.8)	
			4.1% lymphocytes (45.5-54.5)	
			3.3% monocytes (4.5-5.5)	
			0% eosinophils (2.7-3.3)	
		Hemoglobin	13.3 g/dL (11.5-13.5)	
		Platelets	146K/uL (150-350)	
		Sodium	139 mmol/L (135-147)	
		Potassium	2.8 mmol/L (3.4-4.7)	
		Chloride	106 mmol/L (97-107)	
		Bicarbonate	19 mmol/L (17-26)	
		BUN	18 mg/dL (9-22)	
		Creatinine	0.5 mg/dL (0.3-0.6)	
		ALT	12 U/L (9-23)	
		AST	20 U/L (20-40)	
		ALP	224 U/L (132-315)	
		CRP	50.8 mg/dL (<0.5)	
		Total bilirubin	0.9 ng/dL (0.1-0.4)	
		total protein	7.5 g/dL (5.9-7.2	
		Blood culture	No growth*	
	Imaging	CT of the head	No pathology	
		MRI of the head	No pathology	
		MRA of the head	No pathology	
		MRV of the head	No pathology	
5	CSF	Erythrocyte count (RBC)	4643 /uL	Fluid was colorless and cloudy
		Nucleated cells	1217 /uL (<7)	
			5% neutrophils	
			95% lymphocytes	
		Glucose	31 mg/dL (40-70)	
		Total protein	102 mg/dL (40-70)	
		Gram stain	negative	
		PCR**	Positive for *Neisseria meningitidis*	
			Negative for HSV-1	
			Positive for HHV-6	
		Viral culture	No growth	Collected post-antiviral treatment
7	Imaging	MRI of the brain	Small focus of enhancement within or adjacent to the apex of the occipital horn of the right lateral ventricle, possibly involving the ependymal	-
		MRI of the spine (C, T, L)	Enhancement of nerve roots of the cauda equina. Prominent hyperenhancement in the ventral epidural space at the L5-S2 levels, concerning for developing phlegmon or early abscess	
Outpatient follow-up	Blood	Complement levels	Normal	Complement deficiency is associated with increased risk for meningococcal infection
	Genetic testing	Primary immunodeficiency panel	No deficiencies identified	-
	Imaging	MRI of the brain	Persistent focal ependymal/subependymal enhancement of the right occipital horn	-
		MRI of the spine	Persistent inflammation of cauda equina. Prior epidural space finding resolved	-

Empiric meningitic vancomycin dosing and ceftriaxone were started immediately; blood and CSF samples were obtained following antibiotic administration. Dexamethasone was also started around the same time antibiotics were initiated. Emergent computed tomography of the brain with contrast was done prior to lumbar puncture (LP) to rule out contraindication to CSF sampling, which was read as reassuring (Table 1). An LP was immediately done, and CSF output was noted to be cloudy. CSF microscopic analysis revealed significant pleocytosis of 13,500 total nucleated cells (normal (N) 0-7 cells/uL) with 96% neutrophils, hypoglycorrhachia of <10 mg/dL (N 40-70 mg/dL), total protein of 421 mg/dL (N 15-45 mg/dL), and erythrocytes (RBC) of 5750 cells/uL (N 0 cell/uL). A Gram stain of the CSF showed Gram-negative diplococci. Molecular testing of the CSF was positive both for *N. meningitidis* and HSV-1. Acyclovir was immediately added to his regimen.

The patient’s clinical course worsened acutely on the day of admission, with a deteriorating neurologic status that prompted extensive imaging investigation with magnetic resonance imaging (MRI) with and without contrast enhancement, magnetic resonance angiography, and magnetic resonance venography of the brain to rule out increased neurosurgical emergency. All of these baseline studies were reported as normal.

The patient’s sentinel CSF culture demonstrated growth of *N. meningitidis*. Repeat LP was performed on day 5 of hospitalization to reassess CSF parameters and to determine CSF microbiologic clearance. Subsequent CSF examination showed total nucleated cells of 1217 with a 95% lymphocytic predominance; polymerase chain reaction (PCR) remained positive for *N. meningitidis*, was positive for human herpesvirus 6 (HHV-6), and negative for HSV-1 (Table 1).

**Figure 2:**
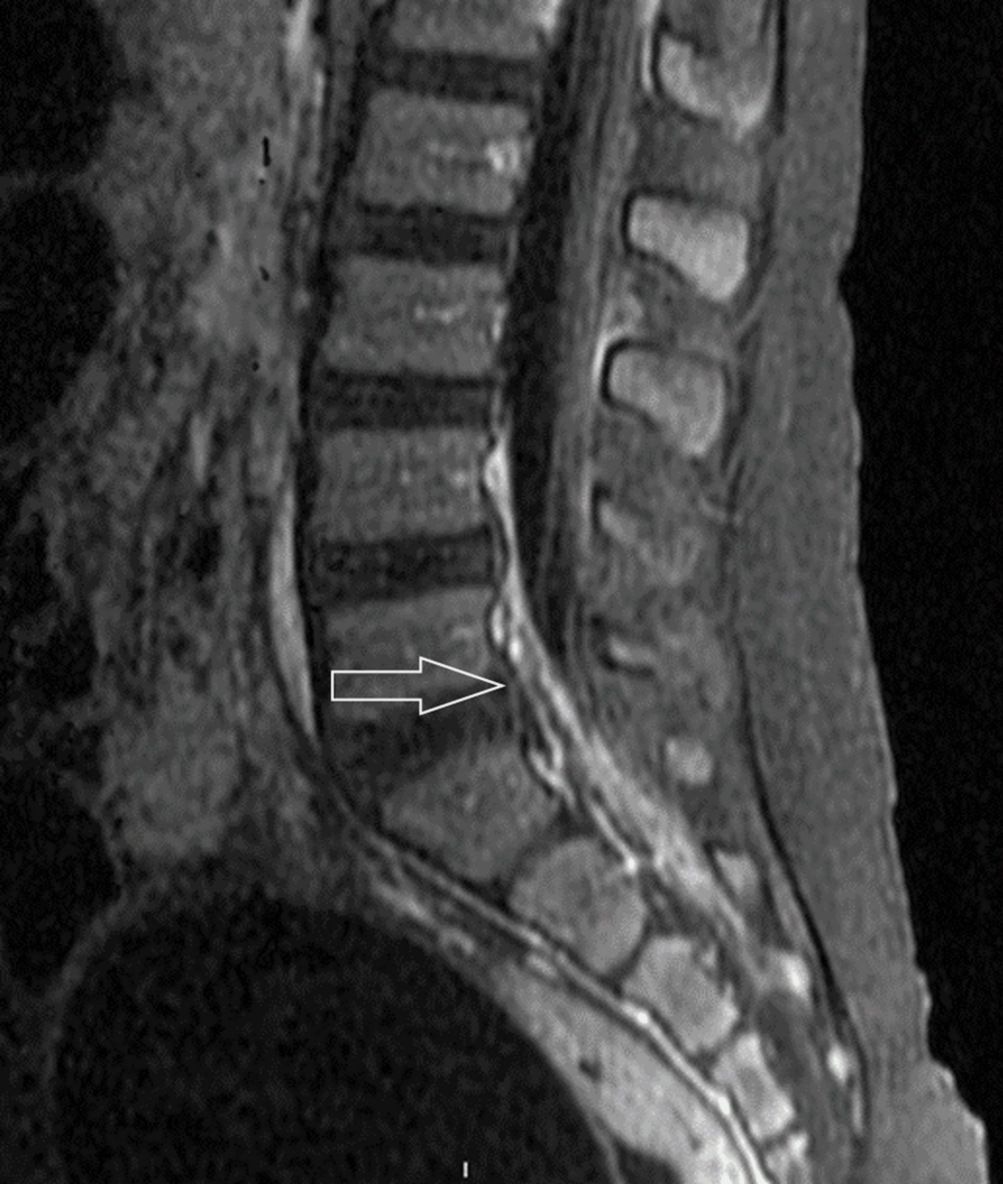
MRI showing prominent enhancement of the ventral epidural space of L5-S1 representing the developing phlegmon (arrow) MRI: magnetic resonance imaging

While the patient demonstrated overall clinical improvement by day 5, gait instability and intermittent fevers to 104.6°F persisted. Brain MRIs and MRIs of the cervical, thoracic, and lumbar spine were obtained after 7 days of hospitalization. The brain MRI showed a new 5 mm focus of hyperintense signal on diffusion either within the atrium of the right lateral ventricle or possibly involving the ependymal surface. There was a subtle focus of enhancement at the apex of the occipital horn of the right lateral ventricle involving the ependyma. This may represent a small purulent material or a microinfarct secondary to meningitis. The cervical spine MRI was negative for findings. The thoracic and lumbar spine MRI showed enhancement of nerve roots of the cauda equina consistent with the patient’s history of meningitis, mild prominence of the upper thoracic region with subtle stranding enhancement representing inflammation or infection, and a prominent hyperenhancement in the L5-S2 level concerning for inflammation, infection, and possible early development of spinal phlegmon (Figure 2).

To evaluate whether the HSV-1 results truly represented a bona fide co-infection, repeat testing of the sentinel CSF sample was performed. Only *N. meningitidis* was positive on the multiplex PCR (BiofireTM, Salt Lake City, Utah) panel. A stand-alone HSV-1 PCR test was negative. Serum HSV 1 and 2 IgG was positive at 13.60, and HSV 1 and 2 IgM was elevated at 0.98 (ref: ≤0.89), suggesting HSV-1 was a bona fide active infection. The patient’s father was later found to have resolving mucosal ulcers in his upper lip that clinically looked like HSV gingivostomatitis. This was swabbed and negative for HSV-1 and HSV-2 by PCR; however, the specimens were obtained on day 11 of his course following a course of acyclovir, implicating theoretically decreased viral load from the sample and a suspected false negative result. The father’s lesions revealed that this has been recurrent, often painful, and most often incited by sunlight exposure, strongly suggesting occult HSV gingivostomatitis.

The patient completed a 21-day course of acyclovir 10 mg/kg every eight hours and a six-week total course of ceftriaxone 50 mg/kg every 12 hours with overall clinical improvement. His surveillance MRI prior to cessation of therapy showed significant resolution of spinal enhancement and overall radiologic findings. He underwent an extensive immune workup, which did not reveal any inborn immunity error or other immunological deficit. He was back to baseline neurologic function on his follow-up after completion of treatment. Hearing evaluation revealed no deficits, but unfortunately, his ophthalmologic exam showed optic neuritis as a complication of his meningoencephalitis that was stable on subsequent ophthalmologic follow-up and did not need additional ancillary intervention.

## Discussion

*N. meningitidis* is a Gram-negative diplococcus responsible for meningococcal disease, ranging from meningitis to disseminated disease [5]. Mortality is 50%, 10-15% even with proper treatment. The development of the purpuric rash in our patient suggests that the presentation was in the early stages of a disseminated disease. Patients aged <1 year, 16-23 years, or >85 years are at greater risk for severe disease, as are those with asplenia, complement deficiency, or HIV [6]. Our patient did not fit into any of these demographics.

A novel aspect of this patient's course is the development of spinal myelitis and phlegmon, which are presumed to be caused by *N. meningitidis* infection. While spinal epidural abscess and myelitis have been reported as complications of *Streptococcus pneumoniae* and *Escherichia coli*, relevant literature is scarce on spinal cord complications of *N. meningitidis* infection [7-11]. Cerebral suppurative complications of *N. meningitidis* are very rare infectious phenomena. While *N. meningitidis* has been reported to produce abscesses in various locations, including the brain, we could not identify any previous reports of spinal abscess/phlegmon associated with the organism.

HSV can cause meningitis or encephalitis. HSV-1 is most commonly associated with encephalitis, while HSV-2 is often associated with meningitis and more often implicated in genital infection [12,13]. Central nervous system infections with HSV-1 are more deadly than those of HSV-2; for HSV-1 encephalomeningitis, the only modifiable risk factor that can affect outcomes is prompt initiation of intravenous acyclovir [3].

A recent study identified mixed bacterial-viral infections as the cause of meningitis in 0.9% of cases [4]. Meningitis due to both meningococcus and another pathogen has been previously reported with various organisms, including *Cryptococcus*, *Bordetella*, and *Streptococcus*, but not HSV (Table 2) [14-17].

**Table 2:** Reported meningitis due to Neisseria coinfection with another pathogen

Coinfection	Organism
Bacterial	Neisseria + Bordetella
	Neisseria + Streptococcus
Viral	*Neisseria + *Enterovirus
Fungal	Neisseria + Cryptococcus

**Figure 3:**
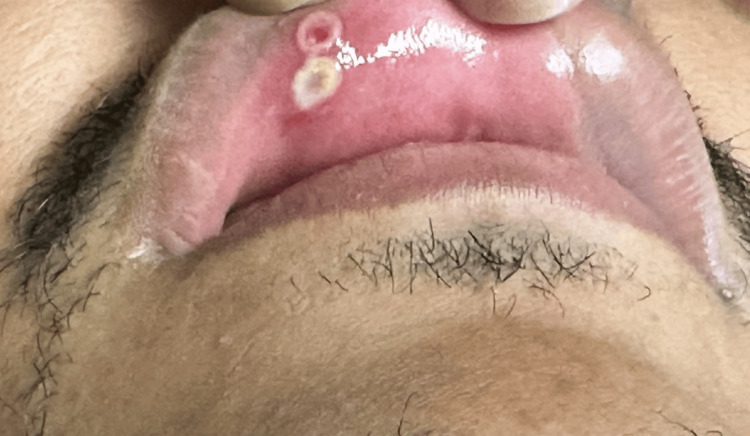
Buccal HSV lesions HSV: herpes simplex virus

The identification of meningococcus by Gram stain, culture, and PCR incontrovertibly establishes the presence of this agent and its role in our patient’s disease. Several factors support the legitimacy of the diagnosis of coinfection with HSV-1. The repeat LP showed a white count of 1200/µL, with 95% lymphocytes, strongly suggesting an ongoing active viral component of the infectious process. The cytology should have theoretically maintained a neutrophilic predominance if it were solely due to bacterial infection. Serological studies demonstrated an elevated HSV IgM, consistent with an acute infection. Following these results, further history attempting to identify a possible source revealed the patient’s father had developed cold sores the week prior to presentation (Figure 3).

Although the presence of the virus was not conclusively demonstrated (viral culture and viral load were not checked as they would add cost to care without altering management), we believe active HSV infection was present. Regardless of whether the HSV component was a reactivation or a new active infection, the sensitive detection by PCR in the CSF is an irrevocable finding that necessitates a cautious clinical approach. Although we cannot confirm this retrospectively, even the minute possibility that active infection was present dictates that clinical management should be sagacious in similar clinical settings. It is tempting to speculate that the unusual development of phlegmon in this case of meningococcus was facilitated by immunosuppression from HSV-1, which is believed to affect bacterial infections in adult intensive care unit patients [18].

A positive HHV-6 PCR on the second LP implicates the question of a triple co-infection, which may be very unlikely. HHV-6 can be chromosomally integrated and is positive in CSF for up to 17% of the population. Therefore, it often has inconsequential implications, especially among immunocompetent patients [19,20]. The HHV-6 was tested twice on hospital day 1 and was negative on both multiplex tests.

Exploration of possible immune compromise showed no evidence of primary immunodeficiency, and complement test results were normal (Table 1).

## Conclusions

We report here what we believe to be potentially the first case in the English language literature of phlegmon in meningococcus meningitis. We also report a case of meningitis due to potential coinfection with HSV-1. With nearly 1% of meningitis cases being due to coinfection, this is a reminder of the importance of continued investigation for potential etiological agents even after one is identified, especially if the additional infectious agent identified might lead to a change in clinical management. The misapplication of Occam’s razor could result in missing potentially lethal contributors to the patient’s illness.
